# Supercharged end-to-side anterior interosseous nerve transfer to restore intrinsic function in high ulnar nerve injury: a prospective cohort study

**DOI:** 10.1186/s12891-024-07650-4

**Published:** 2024-07-20

**Authors:** Mina Abaskhron, Mostafa Ezzat, Andrew Gamal Boulis, Yasser El Safoury

**Affiliations:** Department of Orthopaedic and Traumatology, Kasr Al Ainy Faculty of Medicine, Cairo University, Cairo, Egypt

**Keywords:** Supercharge, Ulnar nerve, Anterior interosseous nerve (AIN), Nerve transfer, Peripheral nerve injury

## Abstract

**Background:**

High ulnar nerve injuries is known to have unfavorable motor outcomes compared to other peripheral nerve injuries in the upper extremity. Functional muscle recovery after peripheral nerve injury depends on the time to motor end plate reinnervation and the number of motor axons that successfully reach the target muscle. The purpose of this study is to assess the functional recovery, and complications following performing supercharge end-to-side (SETS) anastomosis for proximal ulnar nerve injuries. Our study focuses on the role of SETS in the recovery process of high ulnar nerve injury.

**Patient and methods:**

This study is a prospective, single-arm, open-label, case series. The original proximal nerve pathology was dealt with according to the cause of injury, then SETS was performed distally. The follow-up period was 18 months. We compared the neurological findings before and after the procedure. A new test was used to show the effect of SETS on recovery by performing a Lidocaine proximal ulnar nerve block test.

**Results:**

Recovery of the motor function of the ulnar nerve was evident in 33 (86.8%) patients. The mean time to intrinsic muscle recovery was 6.85 months ± 1.3, only 11.14% of patients restored protective sensation to the palm and finger and 86.8% showed sensory level at the wrist level at the end of the follow-up period. Lidocaine block test was performed on 35 recovered patients and showed no change in intrinsic hand function in 31 patients.

**Conclusion:**

SETS exhibit a remarkable role in the treatment of high ulnar nerve damage. SETS transfer can act as a nerve transfer that can supply intrinsic muscles by its fibers and allows for proximal nerve regeneration. We believe that this technique improves recovery of hand motor function and allows recovery of sensory fibers when combined with treating the proximal lesion.

**Trial registration:**

Approved by Research Ethics Committee of Faculty of Medicine- Cairo University on 01/09/2021 with code number: MD-215–2021.

## Background

When comparing proximal ulnar nerve injuries to other peripheral nerve injuries in the upper extremities, the motor outcomes tend to be less satisfactory. The time needed for motor end plate reinnervation and the total number of motor axons that are able to reach the target muscle determine how efficiently a peripheral nerve lesion functionally recover. Hence, acceptable muscle function following these injuries would improve by fast reinnervation of affected muscle fibers and preservation of motor end plate function. Similarly, in cases of severe or recurrent cubital tunnel syndrome with severe motor affection and intrinsic muscle wasting, augmentation of the ulnar intrinsic motor group with additional motor axons would be helpful [1, 2].

In high ulnar nerve (HUN) neuropathy, whether due to traumatic injuries or chronic severe compression, incomplete recovery is expected as the time needed to regenerate the nerve is longer than the motor end plates' life span. HUN injuries are diagnosed when the level of injury is at or higher than the proximal third of the forearm. Supercharge end-to-side (SETS) nerve transfer is a relatively new technique that involves augmenting the ulnar motor branch with the anterior interosseous nerve (AIN) terminal branches. This technique aims at diverting functioning motor fibers of the median nerve from the AIN terminals to the motor baranch of the ulnar nerve (MBUN) in the distal forearm. It is thought that SETS establish a functional anastomosis that acts to “babysit” intrinsic hand function during the regeneration of proximal axons to reach their distal neuromuscular junction. This anastomosis exploits nearby dispensable nerve fibers to shorten the reinnervation time and lead to more favorable functional outcomes [3–5].

The purpose of this study is to assess the functional recovery, and complications following SETS for HUN neuropathies and determine its role in intrinsic muscle recovery.

### Study design, ethics approval and consent

This study is a prospective, single-arm, open-label, case series in which we compare the outcome of proximal ulnar nerve decompression and/or repair before and after SETS without the use of a control group, [6] including adult patients who presented to our Orthopaedic and Traumatology department. Before patients’ recruitment, the Research Ethics Committee (REC) of the Faculty of Medicine, Cairo University approved the study protocol (Code MD-215–2021). The study was conducted under the Declaration of Helsinki [7], and written informed consent was obtained from each patient.

The inclusion criteria allowed enrollment of adult patients with recent HUN neuropathy with preserved AIN function. This includes traumatic injuries and severe compression injuries (cubital tunnel syndromes) that show hand intrinsic muscle weakness or wasting. We excluded patients with ulnar nerve symptoms more than 12 months old or associated with complete AIN injury, patients aged less than 16 years, and patients who refused to participate in the study.

### Patient and methods

All patients were consented, operated upon and followed up between February 2021 to March 2023, 38 patients (mean age 32.5 ± 9.1 years, range 19–51; 25 men = 65.8%) with recent HUN lesions were enrolled in the study at Kar Al-Ainy Cairo University Hospital. Traumatic cases showed evidence of an ulnar motor defect. Compression cases patients show Stage IIB and Stage III according to the modified McGowan classification to ulnar nerve compression [8].

### Preoperative assessment

All patients were assessed by history taking and full physical examination of the upper limb especially wasting, clawing degree by modified Brand’s open hand assessment criteria, Froment test, Wartenberg sign, intrinsic muscle atrophy, sensory testing by monofilament test, and two-point discrimination test. (2PD), Disabilities of the Arm Shoulder and Hand (DASH) score, active and passive range of motion of metacarpophalangeal and interphalangeal joints, grip strength using GRIPX dynamometer, documented Medical Research Council (MRC) grading for intrinsic hand muscles power (finger abduction strength and flexor digitorum profundus (FDP) of little finger strength), nerve conduction studies (NCV) and Electromyography (EMG) studies.

### Operative details

**Fig. 1:**
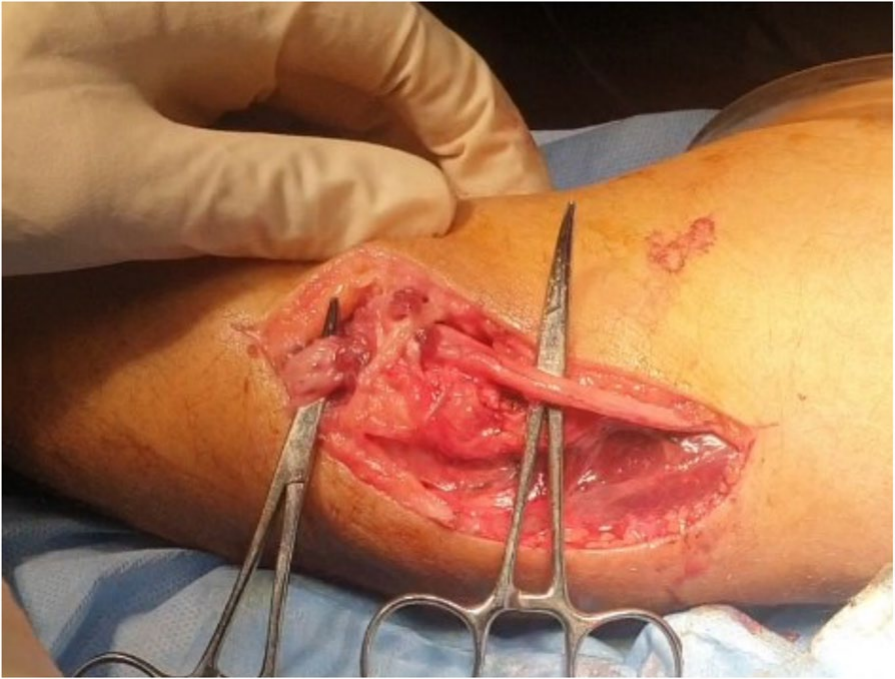
Submuscular anterior transposition of ulnar nerve

**Fig. 2:**
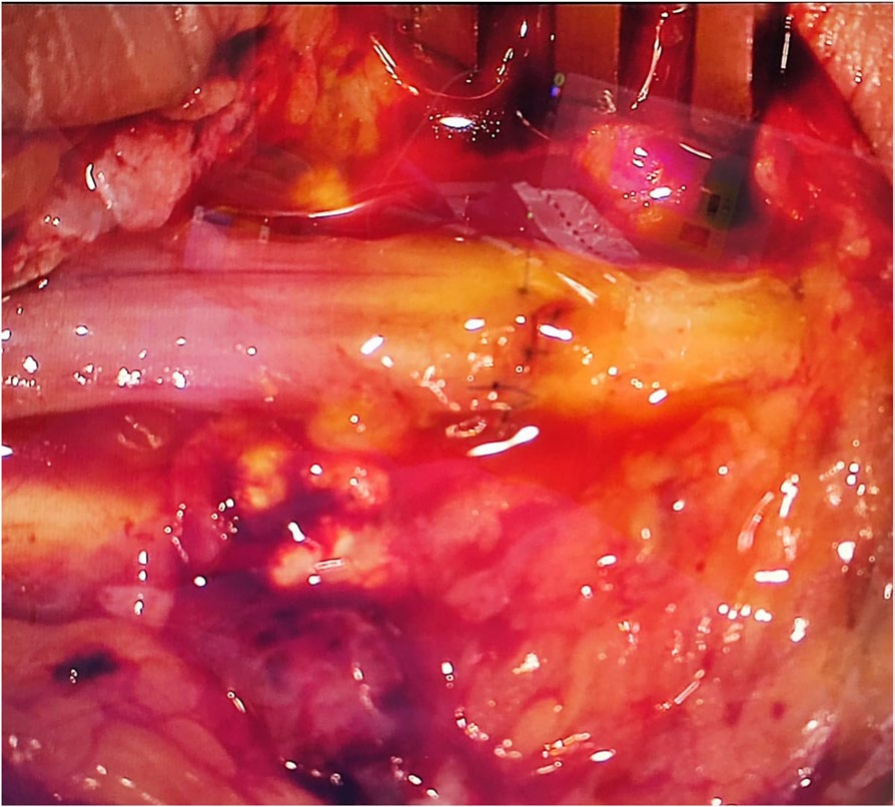
Direct repair of ulnar nerve

In each case, the proximal lesion was addressed initially according to the cause. Anterior transposition of the ulnar nerve (Fig. 1: Submuscular anterior transposition of ulnar nerve) at the elbow level alone was performed on all patients either alone (55%) or with direct repair (Fig. 2: Direct repair of ulnar nerve)(37%) or with graft repair (8%) according to standard practice.

**Fig. 3:**
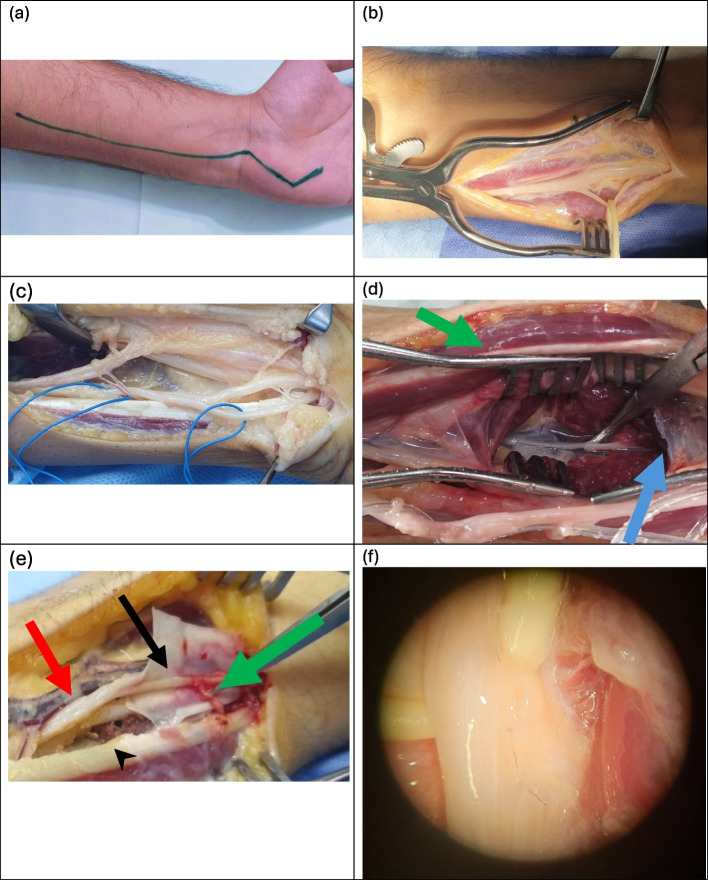
**a** Z-shaped incision, **b** Superficial dissection identification of Dorsal branch of ulnar nerve, **c** Superficial dissection: Anatomical Identification of the ulnar nerve and Guyon canal release, inter-neurolysis with blue tags; Right: MBUN, Left: Ulnar nerve, **d** Deep dissection: Identification of AIN inside Pronator Quadratus, Green arrow: FDS and FDP muscles, Blue arrow: PQ muscle, **e** AIN side-to-end anastomosis with the motor branch of ulnar nerve, Black arrow: MBUN, Green arrow: AIN end-toside anastomosis to MBUN, Red arrow: ulnar nerve proper, Black arrowhead: FCU tendon, **f** SETS done under microscopy

SETS transfer was performed using a Z-shaped incision (Fig. 3(a): Z-shaped incision). The technical aspects of Barbour et al.'s [2] successful distal end-to-side AIN-to-MBUN transfer were followed. The SETS procedure began by identifying the dorsal branch of the ulnar nerve (Fig. 3(b): Superficial dissection identification of Dorsal branch of ulnar nerve). Anatomical identification of the MBUN from the leading edge of hypothenars was done (Fig. 3(c): Superficial dissection: Anatomical Identification of the ulnar nerve and Guyon canal release) and confirmed by intraoperative electric stimulation. The AIN (donor) is then identified (Fig. 3(d): Deep dissection: Identification of AIN inside Pronator Quadratus) and transected at the midportion of the pronator quadratus muscle before it branches, and anastomosed to the side of MBUN (Fig. 3(e): AIN side-to-end anastomosis with the motor branch of ulnar nerve) around 8 cm proximal to the wrist crease under microscopy (Fig. 3(f): SETS done under microscopy). We finally augmented this coaptation with fibrin glue. An above-elbow slab is applied to all patients for 3 weeks.

All patients attended functional rehabilitation sessions, which commenced before surgery supervised by the physiotherapy team. The purpose of this is to determine the baseline status of the involved extremity, educate the patients about the postoperative course of treatment, discuss patient concerns, and address occupational needs and realistic expectations for return to work. Patients received instruction in passive and active range of motion and scar massage. Each patient was assessed individually and rehabilitation continued after the removal of the slab as required.

### Postoperative evaluation

Follow-up appointments were scheduled at 3, 6, 12, and 18 months after surgery. The primary outcomes measured in this study were the recovery of ulnar intrinsic muscle function and the correction of the clawing deformity after surgery. The progression of the Tinel sign and the reported postoperative physical findings in relation to preoperative data were utilized in our evaluation. Wasting is evaluated in comparison to the sound side and with consecutive follow-up photographs. The DASH scores are used to evaluate overall upper limb function. A Lidocaine nerve block test was performed after recovery to assess the contribution of SETS in motor recovery. The test included an ultrasound-guided local injection of 3–5 ml of Lidocaine 2% [9] to the ulnar nerve proximal to the level of SETS. We compared the recovered sensory and intrinsic motor power before and after the injection.

## Results

A total number of 44 patients presented to the Orthopaedic Department of Kasr Alainy Cairo University Hospitals suffering from high ulnar nerve symptoms. Only 40 patients met the criteria of this study (1 patient had associated distal ulnar nerve lesions, and 3 patients had associated AIN/median nerve lesions). Two of the included patients did not meet the minimum required amount of follow-up time. The total number of patients included in this study is 38.

### Patient demographics (Table 1)

**Table 1:** Patient demographics

Variables	Value
Mean age (years)	32.50 ± 9
Diabetes	3 (7.9%)
Hypertension	0
Occupation	
Office worker	22 (57.9%)
Manual workers	16 (42.1%)
Handedness	
Same hand injury	16 (42.1%)
Opposite hand	22 (57.9%)
Smoking	13 (34.2%)

All patients were operated upon between February 2021 to March 2023, 38 patients with a mean age of 32.5 ± 9.1 years ranging from 19–51 years. 25 men were included representing (65.8%) and 13 (34.2%) ladies with recent HUN lesion enrolled in the study at Kasr Alainy Cairo University Hospital.

Three patients (7.9%) were diabetics, and 13 patients (34.2%) were smokers. 16 (42.1%) patients had a HUN lesion to their dominant hand side. Patients were classified according to their occupation as office workers (22, 57.9%) and manual workers (16, 42.1%). According to the level of injury, most of the patients (27, 71.1%) were above the level of the elbow joint. The mean time from injury to SETS was 6.16 ± 3.14 months (Range 0.25–11 months).

### Operative outcomes (Table 2)

**Table 2:** Operative outcome

Variables		Value
Duration of symptoms/injury (months)		6.16 ± 3.14
Level of injury	Distal to the medial epicondyle	5 (13.2%)
	At level of the medial epicondyle	27 (71.1%)
	Proximal to the medial epicondyle	6 (15.8%)
Distance of injury from wrist crease (centimeter)		26.9 ± 2.47
Pathology	compression	16 (42.1%)
	partial injury	9 (23.7%)
	Total injury	13 (34.2%)
Nerve repair without graft		14 (36.9%)
Nerve graft		3 (7.9%)
Anterior transposition of ulnar nerve		38 (100%)
SETS level from wrist crease (centimeter)		7.84 ± 0.71
Recovery incidence		33 (86.8%)
Complication		0
Time to recover (month)		6.85 ± 1.3

The level of nerve injury detected by a clinical wound or EMG was mostly at the level of medial epicondyle (71%). Only 6 patients had the level of injury proximal to the epicondyle and 5 patients were distal to it. The mean distance between the injury site and the wrist crease was 26.9 cm (cm) ± 2.47. Intraoperative identification of the nerve injury type showed that 16 patients (42.1%) suffered from severe compression around the nerve at the cubital tunnel, 13 patients (34.2%) had complete nerve cut and 9 patients (23.7%) had partial nerve laceration with few intact fascicles or neuroma in continuity.

Regarding the proximal nerve injury, intramuscular anterior transposition and nerve decompression were done on all patients. Direct nerve repair without tension was done in 14 patients and only 3 patients required a sural nerve graft (4–5 cables). The level of SETS anastomosis was about 7.84 cm ± 0.71 proximal to the wrist crease.

### Postoperative data (Table 3)

**Table 3:** Post-oeprative data

**Parameter**		**Preoperative**	**Postoperative**	***P*** **-value**
Clawing according to Open-hand assessment of modified Brand’s criteria(%)	Excellent	2.8%	30.6%	< 0.001
	Good	11.1%	41.7%	
	Fair	33.3%	16.7%	
	Poor	52.8%	11.1%	
MRC grade for the strength of finger abduction(%)	Grade 0	57.9%	13.2%	< 0.001
	Grade 1	28.9%	0.0%	
	Grade 2	13.2%	0.0%	
	Grade 3	0.0%	13.2%	
	Grade 4	0.0%	57.9%	
	Grade 5	0.0%	15.8%	
MRC grade for the strength of FDP of little finger (%)	Grade 0	65.8%	13.2%	< 0.001
	Grade 1	13.2%	0.0%	
	Grade 2	0.0%	0.0%	
	Grade 3	15.8%	7.9%	
	Grade 4	5.3%	78.9%	
	Grade 5	0.0%	0.0%	
Grip strength (mean ± SD)		10.63 ± 9.96	23.39 ± 13.84	< 0.001
Positive Froment test (%)		100.0%	13.2%	< 0.001
Positive Cross finger test (%)		21.1%	86.8%	< 0.001
Card tests (%)	R	0.0%	15.8%	< 0.001
	PR	13.2%	44.7%	
	N	86.8%	39.5%	
Positive Wartenberg sign (%)		86.8%	78.9%	0.25
Tinel sign level from wrist crease (mean ± SD)		27.96 ± 3.97	5.86 ± 9.72	
Wasting (%)		94.7%	65.8%	
Electrophysiologic studies (%)	FDI	0.0%	66.7%	< 0.001
	Hypothenars	0.0%	0.0%	––
	FCU	30.6%	5.6%	< 0.001
	FPL&PQ	100%	100%	––
	MUPS	72.2%	72.2%	1
DASH scores (mean ± SD)		46.25 ± 18.24	9.71 ± 10.84	< 0.001
Sensory level (%)	Wrist	100%	78.9%	< 0.001
	Palm	0.0%	13.2%	
	fingers	0.0%	7.9%	

*%* percent of all patients, *R* against resistance, *PR* against partial resistance, *N* negative, *FDI* first dorsal interossei activity, *FCU* flexor carpi ulnaris activity, *FPL* flexor pollicis longus activity, *PQ* pronator quadratus activity, *MUPS* motor unit potentials, *SD* standard deviation

Recovery of the motor function of the ulnar nerve, defined by significant improvement reported between preoperative and postoperative assessment FDI motor activity, was achieved in 33 patients representing 86.8%. The mean time to recovery was 6.85 months ± 1.3. There is no significant difference between the incidence of recovery and the level of injury (*P*-value = 0.441), type of nerve pathology (*P*-value = 0.93), and method of identifying the motor branch (*P*-value = 1). However, recovery was significantly affected when the time between diagnosis and the surgical intervention was above 10 months (*P*-value 0.001).

Close follow-up of other ulnar nerve functions was done including clawing by using the Open-hand assessment of the modified Brand’s criteria, MRC grading of finger abduction muscle strength and flexion strength of the DIP joint of the little finger, grip strength measured by GRIPX Dynamometer, improvement in the Froment test, cross-finger test, Card test, and DASH score. On the other hand, only three patients showed a recovery from positive Wartenburg sign. Despite that 11 patients reported improvement in wasting especially to first web space and interossei, hypothenar wasting was persistent. However, postoperative edema of the limb as a cause of the subjective sense of improvement of wasting could not be excluded in patients who reported early recovery.

The most distal point that shows a positive Tinel sign preoperatively showed a mean distance from wrist crease of 27.96 cm ± 3.97. The mean distance significantly reduced postoperatively to 5.86 cm ± 9.72 (*P*-value < 0.001).

### Sensory assessment at time recovery of motor recovery

Sensation was used as an indicator of axon regeneration proximal to SETS. Sensory recovery was evident in only 5 patients to the palm and 3 patients to fingertips with SMRC scale of S3 or above (functional recovery) at the end of the follow-up period.

### Electrophysiological studies

Regarding neurophysiological studies, FDI was the first muscle to show recovery after 1 year. FCU& FDP showed significant improvement in EMG studies. No improvement was detected in ADM EMG despite the recovery. It is worth mentioning that 5 patients recovered their intrinsic function even though they did not have MUPS in the preoperative studies.

### Lidocaine nerve block test

A new test was developed to detect that the motor recovery was the consequence of SETS procedure impulses rather than from the ulnar nerve itself. We performed this test on 35 patients who had motor ± sensory recovery at or below the level of the wrist. Positive results denoting insignificant change in recovered intrinsic hand power with or without sensory affection after injection were seen in 31 patients (88.5%).

## Discussion

Treatment for high ulnar nerve injuries is extremely challenging [5, 10], and previous studies indicate inadequate recovery of intrinsic muscle function, eventually resulting in functional disability. Surgery to repair proximal nerve lesions produces less favorable outcomes than surgery to correct distal lesions. [11–13]. In cases of high ulnar nerve injury, successful muscle reinnervation depends on sufficient regenerating motor axons reaching the target muscles before degeneration and muscle fibrosis result in the irreversible loss of motor endplates. Axons must travel farther to reach the target organ [14]. There could be several irreversible changes throughout this period that have a negative impact on the result. For proximal injuries at the elbow level, the reinnervation period may last longer than a year [10, 14]. Only 20% of patients in the majority of existing series were found to regain M4 power with proximal nerve surgery alone [12, 13]. Distal nerve transfers enable earlier reinnervation of distal target muscles, therefore improving the chance for intrinsic muscle recovery [15, 16]. Initially, end-to-end motor nerve transfers were carried out to transfer AIN to MBUN [10, 17]. Barbour et al. [2] in 2012 provided the original description of SETS. Many subsequent studies used the approach to demonstrate efficacy in different ulnar nerve pathologies and compare it to traditional treatment.

In our study, 38 patients of similar demographic backgrounds and pathology distribution, underwent SETS procedure in combination with treating the original proximal pathology. 5 out of 8 patients had delayed surgical intervention about 11 months and reported no recovery of their intrinsic hand muscles. We adapted the method described by Barbour et al. While most of the studies used Brunner’s incisions, our study used a Z-shaped incision in the SETS procedure with no reported wrist contracture or wound complication (Fig. 3). No significant difference was noticed in our study regarding the type of proximal nerve surgeries. Preoperative and postoperative outcomes were measured with respect to patient insights, and clinical and electrophysiologic basis.

Regarding clawing assessment, Koriem et al. [18] used Brand’s scoring in the assessment of both claw deformity and disability (metacarpophalangeal and interphalangeal flexion). In our study, we used Brand’s open-hand assessment criteria for simplicity. We also acknowledge patients’ sense of improvement in clawing deformity. A claw splint was not required for any of our patients, which was the same as Baltzer et al. [5]. Thakkar et al. [19] reported an 80% improvement in clawing while our study reported 68%. This may be explained by the short duration of symptoms before surgery in the other study.

As for motor assessment, our study has shown a significant improvement in finger abduction following SETS. FDP strength recovery was crucial in high ulnar nerve injuries to assess the success of the proximal procedure and anticipate proximal nerve regeneration. The Jamar Dynamometer was used in other studies to test grip strength [20, 21]. However, due to lack of availability, we used a Gripx dynamometer (a valid and reliable instrument compared to the Jamar dynamometer [22, 23]) and compared the measures acquired preoperatively vs postoperatively, as well as with affected vs normal side. Combining both results improves the accuracy of detecting recovery of grip strength. Compared to other studies, our study used the Froment test, cross-finger test, card test, and Wartenburg sign for assessment of different muscles for follow-up. Studies showed improvement in the DASH scores which coincides with our findings.

The level of sensory recovery was crucial because it reflects the extent of proximal axon regeneration. Assuming that motor and sensory fascicles of the ulnar nerve regenerate at the same speed, the absence of sensory recovery in cases that achieved intrinsic motor recovery after the SETS procedure was assumed to be a result of AIN regeneration. Only 21% of patients in our study reported sensory recovery to the hand in the first 18 months postoperatively. However, Xie et al. [20] results showed 100% of patients had partial or full resolution of sensory symptoms and 92% showed improvement of muscle wasting. This can be explained by their long-term follow-up period of 28 months. Tinel sign was only utilized in our investigation to track proximal nerve regeneration.

The combined action potentials of all stimulated motor endplates are represented by the compound motor action potential (CMAP) [24]. The absence of CMAPs in denervated muscle, according to the literature, implies that a muscle cannot be reinnervated. The proximity of the ulnar nerve damage, age, sex, or concurrent medical problems did not affect intrinsic muscle healing [25]. Except for EMG done to flexor carpi ulnaris muscle, electrophysiologic examinations in our study indicate no significant change between preoperative and 1-year postoperative assessment despite clinical recovery. Koriem et al. detected reinnervation activity after 12 months, while Xie et al. detected earlier reinnervation activity (6 months). Unexpectedly, Five of the patients who did not have motor unit potentials (MUPS) before surgery exhibited remarkable clinical improvement after SETS.

Despite no complication reported by the end of our study, other studies reported minor complications such as an allergic reaction to wound adhesive, a fungal rash, a hematoma development, persistent elbow pain [21], wound keloid, hypersensitive scar, infection, weakness of forearm pronation strength [20], reflex sympathetic dystrophy [26].

A nerve block test was devised to detect the role of SETS in the recovery of intrinsic function. The injection of the ulnar nerve higher than the level of SETS blocks the proximal nervous signal from reaching the target muscles. After discussing the possible risks of this test, patients agreed to perform the test. No change was seen in 88.5% regarding the MRC grade of recovered motor function after injection. This denotes that the nerve supply of recovered muscle is mainly from SETS transfer. These results coincided with those of another test performed by Dengler et al. who examined the hand intrinsic function in pronation, resisted pronation, and supination, and showed improvement of intrinsic function with resisted pronation was reported in the first 3–9 months after surgery. This was explained by the "donor dominance concept" described by Kahn et al. [27] in 2016 and suggests that intrinsic function is a result of nerve impulses transmitted through AIN. They hypothesized that SETS acts as a supplement to partial recovery till regeneration of proximal ulnar nerve fibers and may act to preserve "Babysit" motor end plates. Our work clarifies that SETS transfer may play a more significant role in recovery.

Our short-term study focuses on the role of SETS in the recovery process of high ulnar nerve injury. Our results did not only support the efficacy of SETS to “babysit” motor end plates but suggested that the new technique can act as a nerve transfer. A possible explanation might be that SETS allow the axons from AIN to grow in the distal ulnar nerve stump and relay nerve impulses from the median nerve to the distal muscles supplied by the ulnar nerve. This was indicated by the shorter time for intrinsic muscle recovery than expected from HUN lesions, the lag of sensory recovery, and the results of the nerve block test. This is important because SETS is a simple procedure and allows faster recovery without disrupting the proximal growing axons. Therefore, SETS has probably superior recovery compared to end-to-end transfer as it preserves more of the proximal motor and sensory axons and allows more motor axons to reach areas supplied by the ulnar nerve.

### Study strength

The study presents comprehensive detailed follow-up outcomes of patients with severe compressive or traumatic HUN lesions including sensory and motor findings. Our results were compared to several studies including recent ones. The introduction of the nerve block test to our follow-up assessment provided new evidence to support our conclusion.

### Limitations

Limitations include a relatively small sample size. Patients who showed earlier recovery of intrinsic muscle function were lost sooner to follow-up because of functional improvement. Since the primary endpoint of this trial was limited to 18-month findings, it is beyond the scope of this study to identify long-term outcomes for SETS. Correction of clawing and muscular wasting can last for more than five years [28]. The neurophysiological studies stated in this article showed little improvement. More sensitive methods to record the regeneration of the ulnar nerve can be discussed in new studies.

These inadequacies imply that the findings of this study should be interpreted with extreme care.

### Further research

Further research is needed to understand the actual role of SETS in the recovery of high ulnar nerve injuries. Since our study was observational, it is impossible to compare other therapy techniques. We suggest research that allows randomization into "repair plus SETS" with "repair only", "SETS only" or "end-to-end nerve transfer" arms. This approach would distinctly show the recovery potential of each approach.

Our study, which focuses on the recovery of motor function, revealed that some muscles did not regain their strength fully. Further studies should consider the need for augmentation of SETS by other traditional tendon transfer techniques to reconstruct intrinsic function or sensory SETS that can be done on the same session of motor SETS or after the failure of recovery.

## Conclusion

SETS exhibits a remarkable role in the treatment of high ulnar nerve damage. The motor recovery of SETS was found independent of the proximal axon regeneration. It allows effective recovery of motor function in a short time that preserves motor end plate and muscle function. It also allows proximal nerve regeneration, which we believe, improves recovery of hand motor function and delivers sensation distally to areas supplied by the ulnar nerve.

## Data Availability

The datasets generated and/or analysed during the current study are not publicly available due to legal/ ethical reasons but are available from the corresponding author on reasonable request.
